# Primary adrenal insufficiency resulting in diagnosis of rare *ABCD1* pathogenic variant in X-linked adrenoleukodystrophy

**DOI:** 10.1210/jcemcr/luag124

**Published:** 2026-05-08

**Authors:** Alison Jin, Anupriya Bhatnagar, Ananda Bryant, Kyaw Soe

**Affiliations:** Department of Internal Medicine, UT Southwestern Medical Center, Dallas, TX 75390, USA; Division of Endocrinology, VA North Texas Health Care System, Dallas, TX 75216, USA; Division of Endocrinology, VA North Texas Health Care System, Dallas, TX 75216, USA; Department of Internal Medicine, UT Southwestern Medical Center, Dallas, TX 75390, USA; Division of Endocrinology, VA North Texas Health Care System, Dallas, TX 75216, USA

**Keywords:** adrenal insufficiency, X-linked adrenoleukodystrophy, *ABCD1*, very long chain fatty acids

## Abstract

X-linked adrenoleukodystrophy (X-ALD) is a rare neurodegenerative demyelinating disorder with variable presentations. We report a case of a 32-year-old African American male with adult-onset X-ALD presenting with primary adrenal insufficiency (PAI) and marfanoid musculoskeletal features. He was initially diagnosed with PAI of unclear etiology and taking only hydrocortisone monotherapy. Further workup revealed negative 21-alpha-hydroxylase antibodies and elevated plasma very long chain fatty acids. Genetic testing confirmed the diagnosis of X-ALD resulting from novel ATP-binding cassette transports sub-family D member 1 gene (*ABCD1*) pathogenic variant. Evaluations for comorbidities by ophthalmology, neurology, and neuropsychiatry were insignificant. Magnetic resonance imaging of the brain and entire spine was unremarkable. There were no cardiac or eye findings to suggest Marfan syndrome and no genetic testing for Marfan syndrome was pursued. This case highlights X-ALD as a potential diagnosis of PAI and the importance of screening plasma very long chain fatty acid levels after ruling out common etiologies.

## Introduction

We report a 32-year-old African American male veteran who was diagnosed with primary adrenal insufficiency (PAI) resulting from an adrenal crisis at the age of 22 years. Initial differential diagnoses for PAI include autoimmune disorder, metastatic malignancy, infiltrative disorder, hemorrhage or thrombosis of the adrenal glands, infectious etiology, medication-induced, and adult-onset X-linked adrenoleukodystrophy (X-ALD). Common etiologies were ruled out clinically. A screening test for X-ALD revealed high levels of very long chain fatty acids (VLCFAs) and genetic testing confirmed the diagnosis of X-ALD with a novel ATP-binding cassette transports sub-family D member 1 gene (*ABCD1*) pathogenic variant.

X-ALD is a congenital neurodegenerative demyelinating disorder that results from a defect in *ABCD1*-producing adrenoleukodystrophy protein [1]. X-ALD has an estimated birth prevalence of 1 in 17 000 when considering both hemizygous males and heterozygous females with no evidence of prevalence variation among regions or ethnicities [1]. X-ALD-associated cerebral adrenoleukodystrophy and adrenomyeloneuropathy (AMN) cause abnormalities in the adrenal cortex and/or nervous system.

The most common presentation is primary adrenal failure, which usually occurs before the age of 15 but can also occur at an older age. The onset of neurological symptoms can be delayed after initial presentation of adrenal failure. Clinical symptoms vary significantly among patients including dementia, weakness, bladder or bowel dysfunction, focal neurologic deficits, and PAI [2]. Elevated VLCFAs are diagnostic along with the identification of hemizygous *ABCD1* pathogenic variant. X-ALD represents a spectrum of phenotypes, which vary in age of onset and severity [2]. Identification of this etiology of PAI will lead to appropriate screening for proband and family members. Timely monitoring of associated endocrinopathies and neurological deficits should be a part of the management [3].

## Case presentation

At the age of 22 years, the patient was hospitalized twice during deployment in Kuwait in 2015 because of complaints of progressive weight loss, lightheadedness, and extreme lethargy. He was diagnosed with acute renal failure resulting from dehydration and was treated with intravenous hydration. He was evaluated by an endocrinologist and the diagnosis of PAI was confirmed by low morning cortisol of 2.9 µg/dL (SI: 79 nmol/L) (reference range, 3-10 µg/dL [SI: 83-276 nmol/L]) and high ACTH of 2342 pg/mL (SI: 515.24 pmol/L) (reference range, 5.9-50.0 pg/mL [SI: 1.3-11.0 pmol/L]). The workup for the etiologies of PAI (adrenal computed tomography, 21-alpha hydroxylase, HIV, tuberculin skin test, and syphilis test) were all normal. Hence, the diagnosis of PAI of uncertain etiology was made. Before the initial presentation, the patient had no physical, learning, or behavioral disabilities. After being diagnosed with PAI, he was started on hydrocortisone (HC) 20 mg in the morning and 10 mg in the afternoon without fludrocortisone. Fludrocortisone was added in May 2019 because of mild hyperkalemia and hypercalcemia (Table 1) without hypotension. He reported no known history of neurological problems or autoimmune disorders, and no known family history of autoimmune disorders, sudden cardiac deaths, or major neurological disorders. The patient's mother is 5 feet 6 inches tall, both his father and brother are 6 feet tall without any major illness. His maternal grandfather was 6 feet tall and died from congestive heart failure at the age of 54 years. The patient is single and has no children.

**Table 1:** Patient laboratory values off hydrocortisone and fludrocortisone (May 2019)

Laboratory test	Value	Reference range
Adrenocorticotropic hormone	2203 pg/mL (486 pmol/L)	6-50 pg/mL (1.3-11.0 pmol/L)
Serum morning cortisol	2.5 µg/dL (69 nmol/L)	3-10 µg/dL (83-276 nmol/L)
Dehydroepiandrosterone sulfate	31 µg/dL (84.12 µmol/L)	85-690 µg/dL (230.7-1872.4 µmol/L)
Aldosterone	<1 ng/dL (<10 pg/mL)	12-196 pg/mL (33.3-543.7 pmol/L)
Plasma renin activity	25 ng/mL/h (6.9 nmol/L/min)	0.3-5.8 ng/mL/h (0.1-1.6 nmol/L/min)
Serum potassium	5.4 mmol/L	3.3-5.1 mmol/L
Serum calcium	10.4-10.8 mg/dL (2.6-2.7 mmol/L)	8.4-10.3 mg/dL (2.1-2.6 mmol/L)
Total testosterone	4.9 ng/mL (17.0 nmol/L)	1.7-7.6 ng/mL [SI: 5.9-26.4 nmol/L]),
LH	7.9 mIU/mL	1.2-8.6 mIU/mL
FSH	4.6 mIU/mL	1.3-19.3 mIU/mL

Abbreviation: SI, Système International units.

## Diagnostic assessment

**Figure 1:**
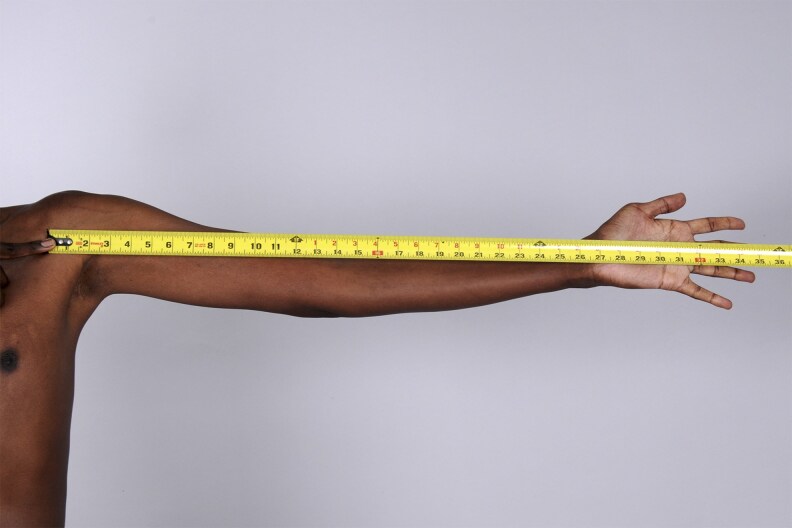
Marfanoid features on examination.

**Figure 2:**
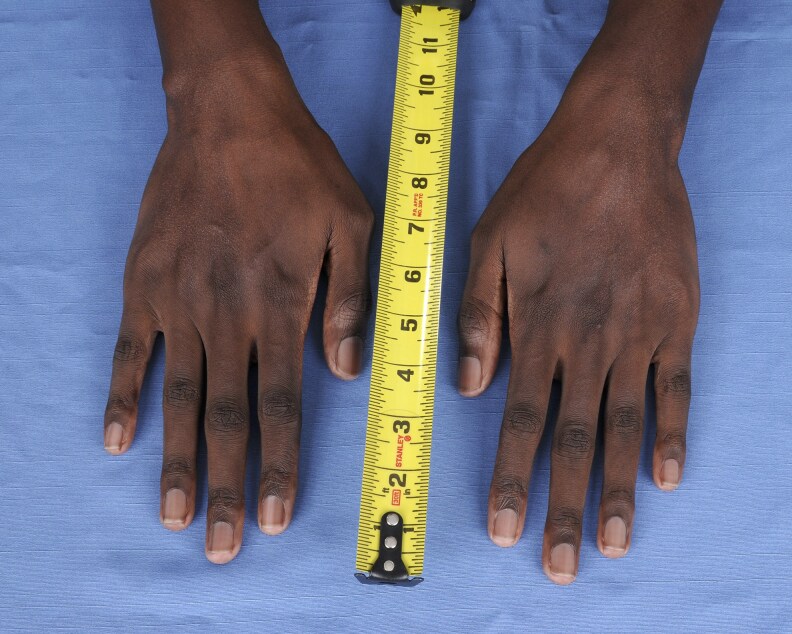
Arachnodactyly.

**Figure 3:**
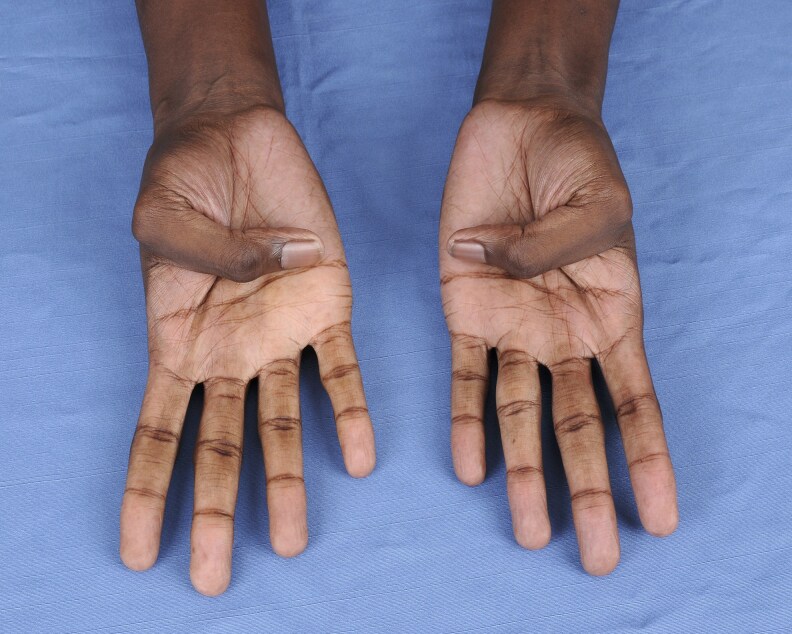
Negative thumb sign.

**Figure 4:**
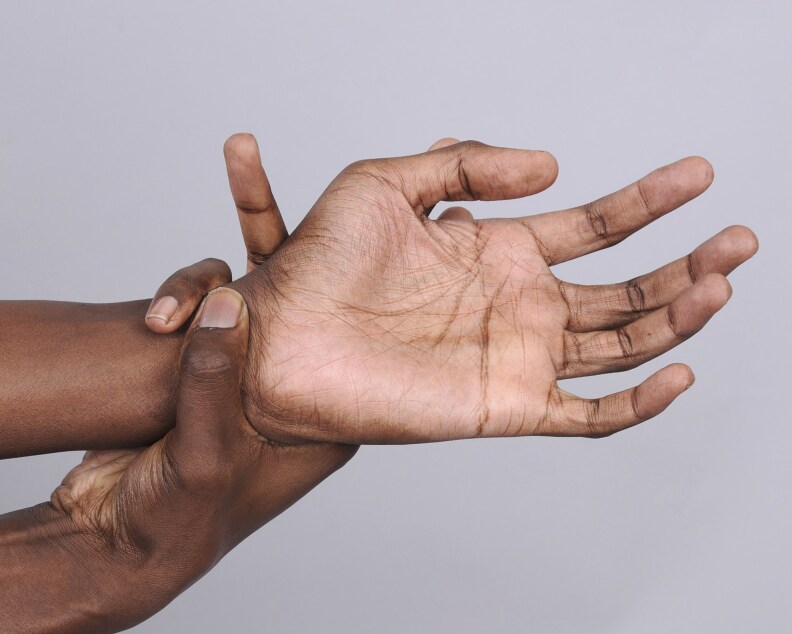
Positive wrist sign.

**Figure 5:**
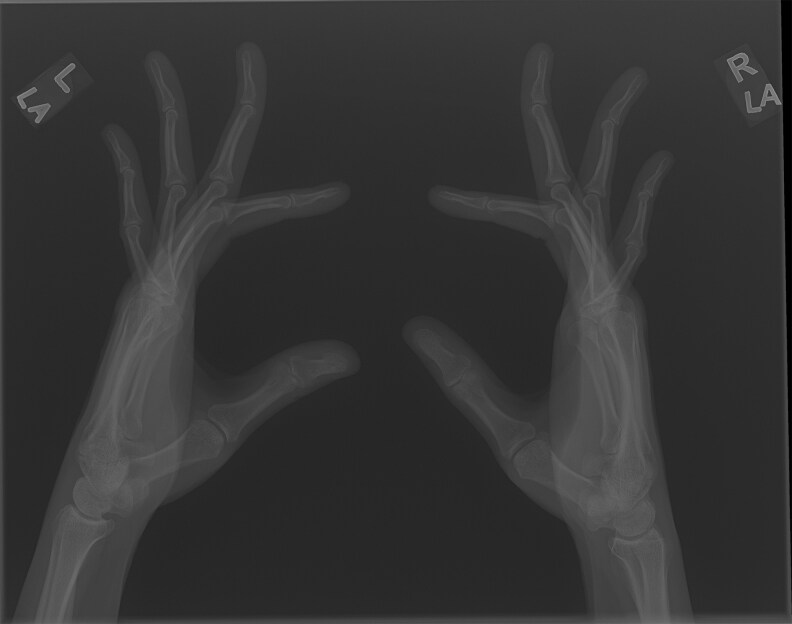
Arachnodactyly of hands on x-ray.

**Figure 6:**
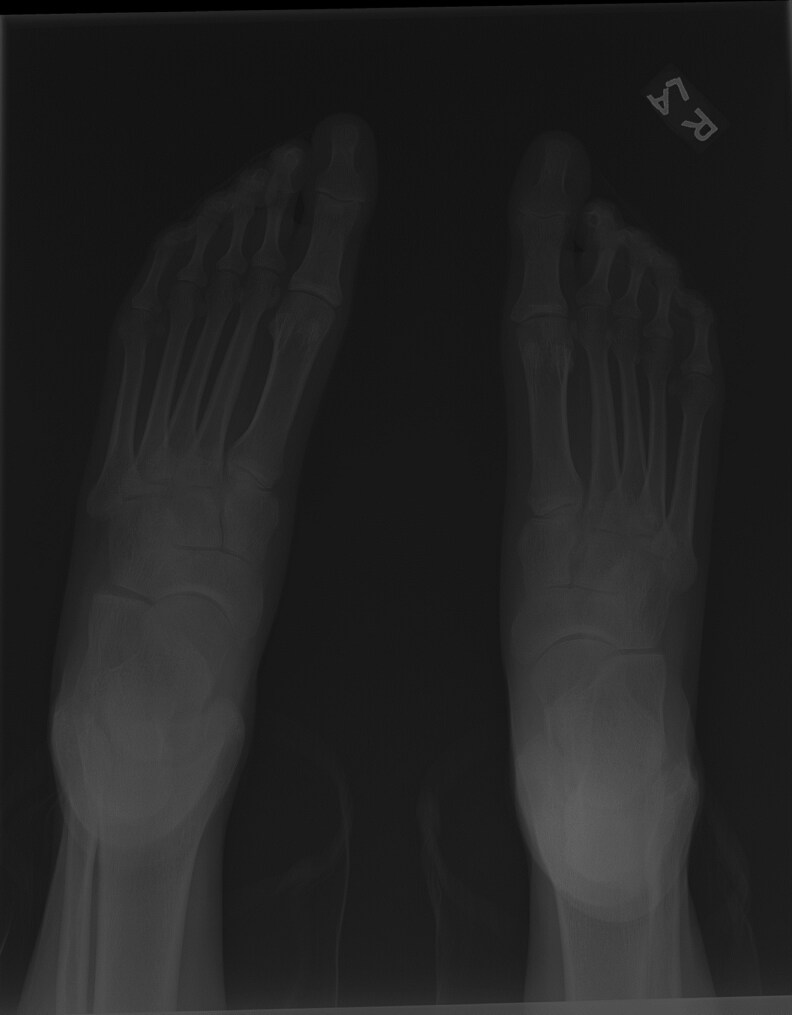
Arachnodactyly of feet on x-ray.

On physical examination, vital signs were unremarkable without dizziness or orthostasis. The patient was noted to have marfanoid features with tall stature and dolichostenomelia (long upper and lower extremities in comparison to the trunk). The Marfan systemic score of the patient per The Marfan Foundation (marfan.org) was 5 (Table 2, Figs. 1-6). A score ≥7 is considered positive for Marfan syndrome. He has no ophthalmological, cardiac, or aortic abnormalities to qualify for the diagnosis of Marfan syndrome. The repeat workup in May 2019 after holding HC for more than 12 hours verified the diagnosis of PAI with high ACTH, low cortisol, low DHEAS, low aldosterone, high plasma renin activity, hyperkalemia, and hypercalcemia (Table 1). Serum electrolytes became normal after fludrocortisone .1 mg per day was initiated and HC was tapered down to 10 mg in the morning and 5 mg in the afternoon. The patient was counseled on steroid sick-day rules and prescribed a medical alert bracelet and emergency HC (Solu-Cortef) injection. Autoantibodies screenings were negative for anti-thyroid peroxidase, anti-glutamic acid decarboxylase, 21-alpha hydroxylase, celiac autoantibodies, and tissue transglutaminase immunoglobulin A and G. Plasma VLCFA were persistently elevated (Table 3). The patient also had normal total testosterone, LH, and FSH with no sexual dysfunction (Table 1).

**Table 2:** Patient's Marfan systemic score

Physical characteristic	Patient	Point contribution
Wrist or thumb sign	Positive	1
Pectus carinatum	Positive	2
Plain flat foot	Positive	1
Scoliosis or thoracolumbar kyphosis	Positive	1
Marfan score		5 (a score of ≥7 is considered a positive systemic score)

**Table 3:** VLCFA values

Very long chain fatty acids	September 2022		February 2023		Reference range
Phytanic acid	1.65 µmol/L		1.52 µmol/L		0.58-2.54 µmol/L
Pristanic acid	0.17 µmol/L		0.19 µmol/L		0.11-0.41 µmol/L
Docosanoic acid, C22:0	74.91 µmol/L		103.11 µmol/L		42.9-112.7 µmol/L
Tetracosanoic acid, C24:0	120.03 µmol/L		159.35 µmol/L		35.6-101.6 µmol/L
Hexacosanoic acid, C26:0	4.69 µmol/L		6.38 µmol/L		0.31-0.81 µmol/L
Ratio C24/C22	1.6		1.55		0.726-0.988
Ratio C26/C22	0.06		0.06		0.0049-0.0118
Ratio pristanic/phytanic	0.11		0.13		0.093-0.254

Abbreviations: C22:0, docosanoic acid; C24:0, tetracosanoic acid; C26:0, hexacosanoic acid.

Genetic testing confirmed the diagnosis of X-ALD resulting from the hemizygous *ABCD1* gene with novel pathogenic variant, defined as c.290A>G due to an amino acid substitution p.His97Arg. There were no neurological complaints. Magnetic resonance imaging (MRI) of the brain and spine showed no remarkable findings. No ectopia lentis by ophthalmology evaluation and no aortic root dilatation or valvular defects by transthoracic echocardiography were noted. Hence, the geneticist decided not to pursue further genetic workup for Marfan syndrome.

## Treatment

The patient has been on HC 10 mg in the morning and 5 mg in the afternoon and fludrocortisone 0.1 mg daily since 2019.

## Outcome and follow-up

Our patient exhibits normal neurological function based on normal neurological examinations by a neurologist and a normal brain and spine MRI. Neuropsychiatry evaluation revealed normal mental faculty, mild to moderate major depressive disorder, and social anxiety disorder. Neurology follow-up with an MRI scan of the brain and spine in 2024 and 2025 remained normal and it was planned to monitor neurological function with MRI and formal neurological examination.

## Discussion

X-ALD is a rare genetic disorder resulting from a defect in the *ABCD1* gene on the Xq28 chromosome, which encodes the adrenoleukodystrophy protein (ALDP). To maintain healthy cellular balance, cells must break down excess VLCFAs in peroxisomes. ALDP is responsible for transporting VLCFAs across peroxisomal membranes for β-oxidation [2]. Pathogenic variant in the *ABCD1* gene leads to dysfunctional ALDP, which in turn results in VLCFA accumulation and apoptosis in the nervous tissue, testes, and adrenal cortex, leading to nerve demyelination, PAI, and hypogonadism [4]. Incidence in US newborns is estimated to be 1 in 17 000 [5]. Age of onset and symptoms vary significantly. High plasma VLCFA levels and genetic testing are helpful in diagnosis though there has been a case of PAI with normal VLCFA levels and subsequent abnormal brain MRI scan [2, 6].

The exact pathophysiology of different phenotypes with variable age of onset among patients with X-ALD remains unknown. There are many pathogenic variants of *ABCD1* and at the time of this manuscript preparation, 1271 unique *ABCD1* variants representing 4500 cases were cataloged by the *ABCD1* Variant Registry. *ABCD1* pathogenic variants have no predictive value for clinical outcomes and no genotype-phenotype correlation. There is no correlation between phenotype, symptoms onset, plasma VLCFA levels, and variants of *ABCD1* [7].

The most common phenotypes of X-ALD include the childhood cerebral form, AMN, and Addison-like presentation. The most severe form is the childhood cerebral form, or cerebral adrenoleukodystrophy, which results in neurological deterioration and PAI in childhood to teenage years [1, 2]. AMN is the most common form of adult-onset X-ALD presenting between ages 20 to 40 years with neurological and reproductive dysfunction [1, 2]. PAI is the most common initial presenting symptom but its penetrance is not well established. It can present in 70% to 80% of males with X-ALD and 5% of female carriers. Isolated PAI (Addison-like presentation) is less common than other phenotypes of X-ALD and accounts for 5% to 10% of patients [2]. Age-dependent risk of PAI is highest until age 10 years and decreases thereafter. However, neurological complications can occur with increasing age [8]. Consequently, X-ALD with PAI-only phenotype is less common among adults [9]. Our patient best fits this rare subtype.

Although our patient has had adult-onset PAI without any neurological defects or mental deficits, the possibility of subsequent emergence of associated comorbidities in a delayed fashion cannot be ruled out. Literature shows long intervals, even decades, between the onset of adrenal insufficiency and neurological symptoms [1].

Our case is the first reported case of adult-onset X-ALD with PAI-only presentation and associated marfanoid musculoskeletal features. This variant c.290A>G (p.His97Arg) has not been reported in any literature or large population database. Because of the convincing clinical features, this variant was interpreted as likely pathogenic. Our patient does not qualify for the diagnosis Marfan syndrome due to a low Marfan systemic score, a diagnostic tool for Marfan syndrome. His score is only 5 and a Marfan systemic score of 7 is needed for diagnosis. The finding of marfanoid features may be coincidental rather than a pathological association. The long-term clinical significance of marfanoid features and this *ABCD1* pathological variant still needs to be determined.

Currently, the cornerstone of treatment for X-ALD is glucocorticoid and/or mineralocorticoid replacement. Not all patients with X-ALD and PAI require mineralocorticoid replacement.

VLCFAs preferentially accumulate in the zona reticularis and zona fasciculata of the adrenal cortex, relatively sparing the zona glomerulosa. Hence, the mineralocorticoid function remains intact in many patients [10, 11]. There are no cases of isolated aldosterone deficiency [8].

Dual-release HC formulation can be used to mimic natural cortisol profile [1]. Dietary modifications including Lorenzo oil and a low-VLCFA diet do not prevent the progression of neurological symptoms and are not recommended therapy [1]. There is no curative treatment with limited treatment options. Stem cell and gene therapies are under investigation because these have demonstrated potential to slow or stop progression of disease with varying success [1, 9].

Diagnosis is challenging because of a lack of genotype-phenotypic correlation, many unknown pathological genetic variants, and unpredictable clinical courses. The liquid chromatography tandem mass spectrometry analysis of C26:0-lysophosphatidylcholine (C26) has been recommended as newborn screening tool of X-ALD in the United States since 2016 [12]. Experts recommend the monitoring and screening of adrenal and neurological functions every few months until the age of 18 years [13]. However, there is no established consensus for monitoring of comorbidities after adulthood, but annual MRI is suggested by experts [9]. X-ALD should be considered as an etiology of PAI among adult males after ruling out common etiologies such as autoimmune and medication-induced.

## Learning points

- X-ALD should be considered a possible diagnosis in males with PAI after ruling out common etiologies because it can be easily overlooked due to delayed-onset neurological deficits.
- Diagnosis can be challenging and delayed because of a lack of genotype-phenotype correlation. Plasma VLCFA levels as a screening test, and subsequent genetic testing for *ABCD1* pathogenic variant are commercially available for diagnosis and family screening.
- Experts recommend newborn screening, and neurological and adrenal function monitoring every few months until the age of 18 years. However, there are no established guidelines or consensus for monitoring among adults because of literature scarcity.

## Contributors

All authors made individual contributions to authorship. A.J. and K.S. were involved in the diagnosis and management of this patient. A. Bhatnagar and A. Bryant were involved in data acquisition and preparation of images. All authors reviewed and approved the final draft.

## Data Availability

Data sharing is not applicable to this article as no data sets were generated or analyzed in current study.

## Abbreviations

ALDP: adrenoleukodystrophy protein
AMN: X-linked adrenomyeloneuropathy
HC: hydrocortisone
MRI: magnetic resonance imaging
PAI: primary adrenal insufficiency
VLCFA: very long chain fatty acid
X-ALD: X-linked adrenoleukodystrophy
